# Analysis of the Physicochemical and Structural Properties of Chestnut Starch After Thermal Processing

**DOI:** 10.3390/foods14071190

**Published:** 2025-03-28

**Authors:** Huijie Fang, Liwen Wang, Yaxi Chen, Zechen Wang, Xianghong Wang, Shuo Wang

**Affiliations:** 1 College of Food Science and Technology, Hebei Agricultural University, Baoding 071000, China; 18822102079@163.com (H.F.); liwenwang@hebau.edu.cn (L.W.); 15933562893@163.com (Y.C.); wangzechen0718@163.com (Z.W.); 2 Tianjin Key Laboratory of Food Science and Health, School of Medicine, Nankai University, Tianjin 300071, China

**Keywords:** dry heat treatment, chestnut starch, digestive properties, structural properties, physicochemical properties

## Abstract

Chestnut is one of China’s traditional export commodities in the international market and enjoys a positive reputation. Its key quality attributes are closely linked to changes in the physicochemical properties of chestnut starch after thermal processing. This study investigated the effects of different temperatures (100 °C, 160 °C, and 200 °C) and times (10, 20, and 30 min) of dry heat treatment (DHT) on the physicochemical and structural properties, as well as the functional properties of chestnut starch. The results demonstrated that DHT increased the solubility (S) and water absorption capacity (WAC), but reduced the swelling power (SP), transmittance, and gelatinization characteristics. DHT modified the starch’s functional properties, increasing its digestibility. This was reflected in the rise in rapidly digestible starch (RDS) and the decline in resistant starch (RS) content. Scanning electron microscopy (SEM) revealed that cracks, crevices, and pores appeared on the starch granule surfaces after DHT. X-ray diffraction (XRD) analyses revealed that the relative crystallinity (RC) of starch decreased with higher temperatures and longer treatment times. The ability of DHT to alter the physicochemical and functional properties of starch provides foundational data for the possible modification of chestnut starch and its application in starch-based food products.

## 1. Introduction

Chestnut (*Castanea mollissima BL*.), also known as hairy chestnut and oil chestnut, is classified as a nut and is often termed as the ‘king of dried fruits’ [1]. Originating in China [2], chestnuts rank among the earliest cultivated nuts in the region, with a history of cultivation spanning at least 2500 years. Chestnuts are rich in starch, protein, fat, vitamins, and minerals [3]. Moreover, chestnuts offer a variety of health benefits, including nourishment of the stomach and spleen, the strengthening of the kidneys and tendons, the improvement of blood circulation, facilitation of blood clotting, and antioxidant properties [4].

Starch, one of the most abundant nutrients in chestnuts, is primarily composed of amylose and amylopectin. Amylose is made up of linear chains of glucose that are connected by α-1,4-glycosidic linkages. In contrast, amylopectin is a branching polymer made up of glucose units connected by α-1,4- and α-1,6-glycosidic linkages [5]. Amylopectin exhibits a helical conformation and possesses a greater molecular weight compared to amylose. Starch can be categorized into three groups according to enzymatic digestion rates: rapidly digestible starch (RDS), slowly digestible starch (SDS), and resistant starch (RS) [6]. Decreasing the RDS content or enhancing SDS and RS proportions helps to regulate postprandial blood glucose levels, reducing the likelihood of diabetes and its associated complications [7]. However, the limitations of natural starch regarding viscosity, gel strength, solubility, and digestibility adversely affect its functionality, efficiency, and product quality in the food industry, potentially restricting its applications [8]. Therefore, modifying starch is essential to fulfill specific industrial needs.

Dry heat treatment (DHT) is a widely utilized method for physical starch modification, designed to reduce the moisture content of starch and partially denature its molecular structure [9]. Compared with other modification methods, DHT enhances starch performance without the use of chemical substances, making it an environmentally friendly approach [10]. DHT has demonstrated significant potential in improving the physicochemical properties of natural starches. The impact of DHT on starch’s physicochemical and structural properties varies based on its source, treatment time, and temperature [11]. These effects are mainly observed in changes to starch granule morphology, crystallinity, and rheological properties [12], as well as amylose content, solubility, swelling power, water absorption capacity, viscosity, and viscoelasticity [13]. At a lower DHT temperature (120 °C) for 2 h, starch molecular chains decompose, leading to a reduction in molecular weight, which can affect digestibility [14]. Continuous or cyclic heating for 3–15 h at a moderate temperature (180 °C) reduces the relative crystallinity, short-range ordering [15], gelatinization temperature, and enthalpy [16], while increasing starch resistance. At higher treatment temperatures (190 °C) for 2 h, amylopectin undergoes significant structural transformations, with long chains breaking into shorter chains and forming soluble branched amylopectin. DHT effectively alters the functional properties of starch. Most DHT studies have concentrated on rice [17], maize [18,19], and potato starches [20,21,22,23], with limited research available on chestnut starch, especially regarding the effects of DHT at varying temperatures and times.

Most studies on dry heat treatment (DHT) typically involve durations ranging from several to ten hours, reflecting a relatively lengthy process. However, in the practical thermal processing of chestnuts, the heating time is approximately 20 min [24]. Based on this, the present study designs DHT on chestnut starch under three temperature conditions commonly encountered during boiling (100 °C), frying (160 °C), and roasting (200 °C), with treatment times of 10, 20, and 30 min. The goal was to investigate the effects of DHT on the physicochemical and functional properties of chestnut starch under realistic processing conditions. Additionally, the study aims to expand the potential applications of DHT-modified starch and provide valuable data for understanding quality changes in chestnuts under practical thermal processing conditions.

## 2. Materials and Methods

### 2.1. Materials

Yanshanzaofeng was picked in Chengde, Hebei, China. Porcine pancreatic α-amylase (5 U/mg) and the amylose content kit (No. AC10666) were purchased from ACMEC Biochemical Technology Co., Ltd. (Shanghai, China). Amyloglucosidase (100,000 U/mL) was obtained from Yuanye Biotechnology Co., Ltd. (Shanghai, China), while the glucose oxidase kit (GOPOD) was procured from Megazeme Co. (Shanghai, China). All other chemicals used in this study were of analytical grade.

### 2.2. Sample Preparation

Briefly, chestnuts were hulled, sliced, and subsequently freeze-dried using a vacuum freeze-dryer. The dried chestnuts were then ground using a grinder and passed through an 80-mesh sieve. Freeze-dried chestnut powder was immersed in NaOH solution (0.2%, *w*/*v*) at a 1:2 (*w*/*v*) ratio and soaked overnight [25]. The resulting slurry was filtered through a 200-mesh sieve to remove residues. The filtrate was then centrifuged at 4000× *g* for 5 min to separate the supernatant and discard the brown upper layer. After repeatedly washing the precipitate with distilled water, the supernatant was centrifuged until it turned transparent. It was then further washed with anhydrous ethanol until the supernatant became clear, after which the supernatant was decanted. The obtained starch was then dried in an oven at 40 °C for 24 h.

Chestnut starch was heated at 100 °C, 160 °C, and 200 °C for 10 min, 20 min, and 30 min, respectively, and subsequently cooled to room temperature. These samples were named SB-10/20/30, SF-10/20/30, and SR-10/20/30, while the untreated raw chestnut starch was designated as CS.

### 2.3. Color Characteristics

The color difference in the processed chestnut starch was measured using a colorimeter (LS173, Shenzhen Linshang Technology Co., Ltd., Shenzhen, China.), and the L*, a*, and b* values were recorded. The color difference (ΔE) was calculated using the following formula:

(1) $$∆E=\sqrt{\left({\left(∆L^{*}\right)}^{2}+{\left(∆a^{*}\right)}^{2}+{\left(∆b^{*}\right)}^{2}\right)}$$

### 2.4. Amylose Content

An amylose content kit (Item No. AC10666) from ACMEC Biotechnology Co., Ltd. (Shanghai, China) was used to measure the amylose content.

### 2.5. Solubility and Swelling Power Messurement

In a water bath, 30 mL of 2%, *w*/*v* starch solution was heated to 80 °C for 30 min while being constantly stirred. Once it had cooled to an ambient temperature, it was centrifuged for 15 min at 4000× *g*. After 2 h of drying at 105 °C, the supernatant was weighed. The values of solubility (S) and swelling power (SP) were calculated using the following formula:

(2) $$S(\%)=(W_{1}/W_{0})\times 100$$

(3) $$SP(g/100\;g)=W_{2}/[W_{0}\times (100-S)]\times 100$$

where W_0_: weight of starch; W_1_: weight of dry supernatant; and W_2_: sediment weight.

### 2.6. Transmittance

50 mL of starch suspension (1%, *w*/*v*) was heated in a water bath at 100 °C for 15 min while being continuously stirred. The absorbance was measured at a wavelength of 620 nm using a UV spectrophotometer (Thermo Scientific, Waltham, MA, USA) after the suspension had cooled to room temperature.

### 2.7. Water and Oil Absorption Capacity

According to the method of Liu et al., the sample preparation method was slightly modified [26]. First, 1 g of chestnut starch was homogeneously dispersed in 10 mL of distilled water or corn oil. After 30 min of vortexing at room temperature, the mixture was centrifuged for 10 min at 4500× *g*. Centrifugation was followed by the decantation of the supernatant. The water absorption capacity (WAC) and oil absorption capacity (OAC) were determined using the following formula:

(4) $$WAC(g/g)=(W_{2}-W_{1})/W_{0}$$

(5) $$OAC\left(g/g\right)=(W_{2}-W_{1})/W_{0}$$

where W_0_: weight of the sample; W_1_: weight of the sample and the centrifuge tube; and W_2_: weight of the sediment and the centrifuge tube.

### 2.8. Retrogradation Property Messurement

The method was adapted from Shi et al. with slight modifications [27]. A 1% (*w*/*w*) starch suspension was allowed to cool to room temperature following 30 min of gelatinization at 100 °C. The suspension was transferred to a 100 mL volumetric flask and allowed to stand at room temperature. At regular intervals, the supernatant’s volume was measured and noted. The retrogradation was calculated using the following formula:

(6) $$Retrogradation\left(\%\right)=V_{1}/V\times 100$$

where V: total volume of starch paste and V_1_: supernatant volume.

### 2.9. Digestive Properties Messurement

100 mg of starch (dry weight), 4 mL of 0.1 M sodium acetate buffer (pH 5.2), and 1 mL of a porcine pancreatic α-amylase and amyloglucosidase mixture were thoroughly mixed. The suspension was then placed in a 37 °C constant temperature water bath. Aliquots were removed at 20, 40, 60, 80, 100, and 120 min, respectively. To stop the enzymatic reaction, 0.9 mL of ethanol was mixed with 0.1 mL of the hydrolysis product sample. The GOPOD method was used to measure the amount of glucose present in the hydrolysate. The glucose concentration in the hydrolysate was determined using the GOPOD method. The content of RDS, SDS, and RS was calculated using the following formula:

(7) $$RDS(\%)=(G_{20}-FG)\times 0.9$$

(8) $$SDS(\%)=(G_{120}-G_{20})\times 0.9$$

(9) $$RS(\%)=100-\left(RDS+SDS\right)=100-(G_{120}\times 0.9)$$

where G_0_: amounts of glucose at 0 min; G_20_: amounts of glucose at 20 min; G_120_: amounts of glucose at 120 min; and FG: amounts of free glucose.

### 2.10. Thermal Property Messurement

Differential scanning calorimetry (DSC 3+, METTLER TOLEDO, Hong Kong, China) was used to analyze the thermal properties of starch, following the method of Chandak et al. (2024) [28]. The sample powder (3 mg) was added to deionized water (1:3, *w*/*v*), and the blank was an empty pot. The temperature range was set between 20 °C and 120 °C with a heating rate of 10 °C per min. The gelatinization temperature range (R) was calculated using the following formula:

(10) $$R=T_{C}-T_{O}$$

where T_o_: onset temperature and T_c_: conclusion temperature.

### 2.11. Scanning Electron Microscope (SEM)

After using conductive adhesive tape to bind the starch samples to an aluminum sheet, they were sprayed with gold. The microstructures were photographed using a Zeiss Sigma 300 scanning electron microscope (Carl Zeiss, Oberkochen, Germany) at 1000× and 4000× magnifications.

### 2.12. Fourier Transform Infrared Spectroscopy (FT-IR)

FTIR analysis was performed using a Fourier transform infrared spectroscope (Thermo Nicolet iS5, Waltham, MA, USA). KBr and starch samples were crushed in a 1:100 ratio and then pressed into flakes. The spectral scanning range was 4000–400 cm^−1^, with 64 scans and a resolution of 4 cm^−1^.

### 2.13. X-Ray Diffraction

Samples of starch were examined for crystal structure using X-ray diffraction (Rigaku Corporation, Tokyo, Japan). With a wavelength of 0.1542 nm, Cu-Kα radiation served as the X-ray source. The scanning range was set at 5 to 50° with a scanning speed of 2°/min, and the voltage and current were 40 mA and 40 kV, respectively. MDI Jade 6 software was used to determine the starch samples’ relative crystallinity (RC).

### 2.14. Statistical Analysis

IBM SPSS Statistics 23 was used for data analysis, and all experiments were carried out in triplicate. The mean ± standard deviation is used to express the data in the table. Additionally, a one-way analysis of variance (ANOVA) was used to perform Duncan’s Multiple Range Test (DMRT) in order to identify significant differences between means (*p* < 0.05). Origin 2021 Pro was used to plot the data.

## 3. Results and Discussion

### 3.1. Color Difference Analysis

**Figure 1:**
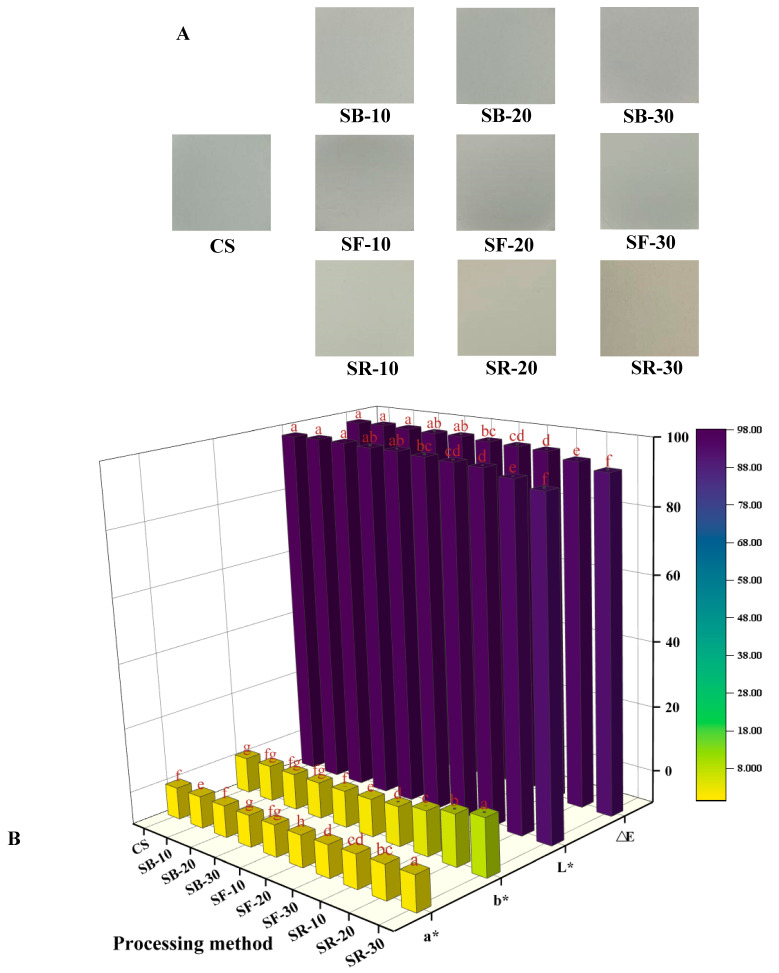
Image of chestnut starch (**A**), the color variation in chestnut starch (**B**) after dry heat treatment. The different letters in the picture are significantly different (*p* < 0.05). CS, chestnut starch; SB-10/20/30, simulate boiling (100 °C) for 10 min, 20 min, and 30 min; SF-10/20/30, simulate frying (160 °C) for 10 min, 20 min, and 30 min; and SR-10/20/30, simulate roasting (200 °C) for 10 min, 20 min, and 30 min.

The color difference (∆E) value is commonly used to reflect variations in color. The L* value, where 0 indicates black and 100 indicates white, is used to represent lightness. The a* value represents the red–green axis, where green is represented by negative values and red by positive values. The b* value represents the yellow–blue axis, with positive values denoting yellow and negative ones denoting blue. From Figure 1A, it can be seen that the color of CS is white and brighter. After thermal processing, the starch colors of the SB, SF, and SR groups showed a tendency to become darker and yellower. As seen in Figure 1B, the L* and ∆E values of the SB group were not significantly different from those of CS; the L* and ∆E values of the SF group were reduced. The SR group’s L* and ∆E values were considerably lower (*p* < 0.05), suggesting that heat processing darkened the color of CS, which is in agreement with the results of Figure 1A. With the increase in the thermal processing temperature, the a* value gradually changed from negative to positive. Compared with CS, the b* values of the SB, SF, and SR groups increased, indicating that the color of chestnut starch tended to become yellow after thermal processing, which was in line with Figure 1A’s findings. The breakdown of starch molecules during DHT could be the cause of this. During this process, gelatinization and caramelization may occur to form brown compounds, thereby reducing the whiteness of starch [29].

### 3.2. Amylose Content and Transmittance

**Figure 2:**
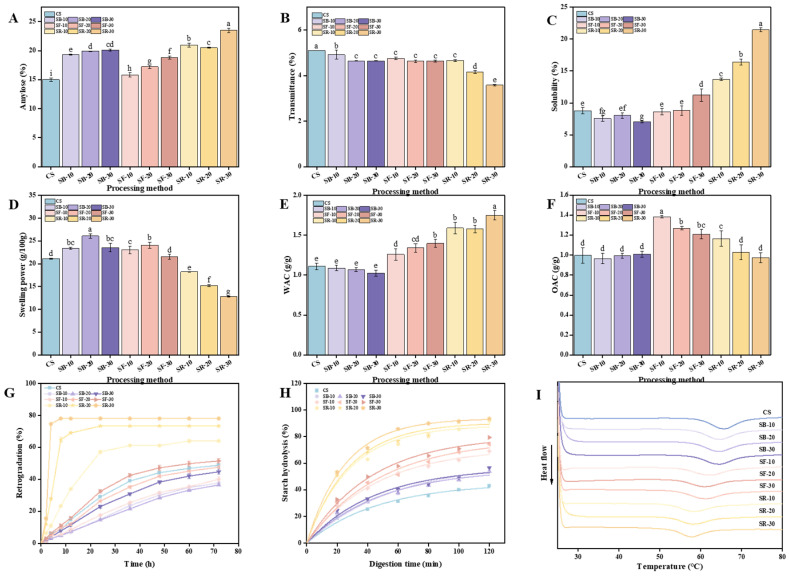
Amylose content of chestnut (**A**); transmittance (**B**); solubility (**C**); swelling power (**D**); WAC (**E**); OAC (**F**); retrogradation (**G**); starch hydrolysis (**H**); and differential scanning calorimetry (DSC) curve (**I**) of chestnut starch after dry heat treatment. The different letters in the pictures are significantly different (*p* < 0.05) CS, chestnut starch; SB-10/20/30, simulate boiling (100 °C) for 10 min, 20 min, and 30 min; SF-10/20/30, simulate frying (160 °C) for 10 min, 20 min, and 30 min; SR-10/20/30, simulate roasting (200 °C) for 10 min, 20 min, and 30 min; WAC, water absorption capacity; and OAC, oil absorption capacity.

The physicochemical and nutritional characteristics of starchy foods are critically influenced by amylose. As shown in Figure 2A, the amylose content in chestnuts increased significantly (*p* < 0.05) after DHT, reaching its highest value of 23.53% in the SR group treated at 200 °C, which aligns with the results reported by Zhang et al. and Hui et al. Zhang et al. pointed out that during DHT, amylopectin molecular chains are prone to breaking. These fragmented chains can rearrange into new amylose structures, thereby increasing the amylose content [11] Hui et al. pointed out that the increase in amylose content is due to the disruption of the integrity of starch granules by DHT, which triggers the breakdown of amylopectin chains, leading to the formation of new amylose chains [30]. Additionally, breaking the hydrogen bonds between amylose and amylopectin could increase the affinity for iodine binding, further contributing to the increase in amylose content [12]. As illustrated in Figure 2B, the transmittance of CS decreased significantly (*p* < 0.05) after DHT, reaching its lowest value of 2.58% in the SR group treated at 200 °C. The transmittance of the SB group declined as heat treatment time increased, while the SF group showed statistically insignificant changes. In contrast, the SR group exhibited significant (*p* < 0.05) reductions in transmittance with longer thermal processing times. The transmittance ranking among the three groups post DHT was SR > SF > SB, indicating that transmittance decreased as the heat treatment temperature increased. DHT-induced morphological changes in starch granules result in inhomogeneous surfaces characterized by holes and grooves, enhancing light scattering and thereby reducing transmittance, which is consistent with the findings reported by Yang et al. [31]. Furthermore, DHT can induce starch rupture and elevate the short-chain amylose content. These short chains can recombine into reticulated structures, leading to turbid starch pastes with diminished light transmission, which is consistent with the findings reported by Gu et al. [32]. These changes upset the starch granules’ orderly structure, reducing their light transmission capabilities.

### 3.3. Solubility and Swelling Power

Solubility (S) and swelling power (SP) are fundamental physical properties of starch, influenced by different factors including temperature, starch granule size, starch type, and amylose content. The temperature and time of DHT exhibited distinct effects on the S and SP of chestnut starch (Figure 2C,D). As illustrated in Figure 2C, the S of chestnut starch exhibited a general increasing trend with rising heat treatment temperatures after DHT, which is consistent with the findings reported by Gautam et al. [33]. In comparison to CS, the S of chestnut starch decreased under the lower heat treatment temperature SB (100 °C). For heat treatment times of 10 and 20 min (SF-10/20) at a moderate temperature (160 °C), there was no significant difference in S compared to CS. However, a significant increase (*p* < 0.05) in S was observed at a heat treatment time of 30 min (SF-30). Under higher heat treatment temperatures (200 °C), the S increased significantly (*p* < 0.05) with extended heat treatment times. This increase could be explained by the production of shorter amylose chains as a result of the DHT-induced cleavage of starch molecular chains, especially long-chain amylopectin molecules. The enhanced exposure of molecular chain ends may further contribute to increased S [11]. Partial disruption of the amorphous region during DHT could lead to amylose leaching and increased S [34]. As shown in Figure 2D, the SP of chestnut starch demonstrated an overall trend of increasing and then decreasing with rising heat treatment temperatures relative to CS. The SP significantly increased (*p* < 0.05) under the lower heat treatment temperature SB (100 °C). At a moderate heat treatment temperature (160 °C), the SP of chestnut starch increased significantly (*p* < 0.05) for times of 10 and 20 min (SF-10/20). However, no significant differences were observed for a 30 min time (SF-30). At higher heat treatment temperatures (200 °C), the SP decreased significantly (*p* < 0.05) with prolonged heat treatment times. This decrease may result from the agglomeration of starch molecules at elevated temperatures, which impairs SP [35]. Due to the rearrangement of the crystalline regions of starch induced by high temperatures, the degree of bonding between starch chains increases, hindering the diffusion of amylopectin molecules and resulting in a reduction in SP [34]. In their study on sweet potato starch, Gou et al. found that after DHT, the interactions between amylose and amylose-amylopectin chains may be enhanced. This could lead to a reduction in the diffusion capacity of amylopectin and amylose molecules, thereby decreasing the SP [23].

### 3.4. Water and Oil Absorption Capacities

The water and oil absorption capacities (WAC and OAC) of starch are fundamental physical properties impacted by the intrinsic starch structure and external processing conditions. The temperature and time of DHT exhibited distinct impacts on the WAC and OAC of CS (Figure 2E,F). As illustrated in Figure 2E, the WAC of chestnut starch showed a consistent upward trend after DHT. Under the lower heat treatment temperature SB (100 °C), the WAC of chestnut starch did not show significant differences compared to CS. However, at the medium heat treatment temperature SF (160 °C), the WAC increased significantly (*p* < 0.05) relative to CS and continued to increase with extended heat treatment times. At the higher heat treatment temperature SR (200 °C), the WAC also increased significantly (*p* < 0.05) compared to CS, reaching a peak value of 1.75 g/g after 30 min of heat treatment (SR-30). This implies that as heat treatment temperatures increased, the WAC of CS tended to rise. This observed increase may result from the breakdown of starch into soluble sugars during DHT, enhancing WAC [36]. Additionally, during the DHT process, some hydrogen bonds between the amorphous and crystalline regions of starch are broken, leading to a slight expansion of the amorphous regions. These changes enhance the hydrophilicity of the starch molecules and contribute to the higher water absorption capacity observed in the DHT-modified samples [37].

As shown in Figure 2F, the OAC of chestnut starch did not show significant differences at the lower heat treatment temperature SB (100 °C) compared to CS. At moderate to high DHT temperatures, the oil absorption capacity (OAC) showed an increasing trend, which is consistent with the findings reported by Liu et al. in their study on barley starch at 150 °C and 180 °C [38]. At the medium heat treatment temperature SF (160 °C), the OAC increased significantly (*p* < 0.05) compared to CS, but gradually declined with extended heat treatment times. At the higher heat treatment temperature SR (200 °C), the OAC decreased with extended heat treatment times. While the OAC of SR-10 was substantially greater than that of CS, no significant differences were observed for SR-20 or SR-30. On the one hand, starch granules’ surfaces are disturbed by DHT, which results in the development of pores and fissures, thereby increasing the specific surface area and enhancing water- and oil-holding capacities. On the other hand, a change in the crystal structure of the starch may enlarge the central cavity within the granules, enhancing their capacity to adsorb water and oil [39].

### 3.5. Retrogradation Property

The retrogradation property of starch refers to the phenomenon where starch molecules recombine through hydrogen bonding during the cooling process after pasting, leading to the formation of a gel or precipitate with an ordered structure. A smaller volume of the supernatant after natural sedimentation suggests weaker starch retrogradation, as it reflects less phase separation and a more stable starch paste system [40]. The temperature and time of DHT have distinct impacts on the retrogradation of chestnut starch (Figure 2G). As shown in Figure 2G, retrogradation generally exhibited an increasing trend with rising heat treatment temperatures. Compared to CS, the retrogradation of chestnut starch showed a decreasing trend at the lower heat treatment temperature (SB, 100 °C). Specifically, the retrogradation of SB-10 was generally lower than that of SB-20 during the first 12 h of standing. However, as the standing time increased, the retrogradation of SB-10 exceeded that of SB-20 between 24 and 72 h. Among the SB groups, SB-30 displayed greater retrogradation across the 0–72 h timeframe than both SB-10 and SB-20. At the moderate heat treatment temperature (SF, 160 °C), retrogradation progressively increased with extended heat treatment times. While the retrogradation of SF-10 and SF-20 was lower than CS, SF-30 exhibited greater retrogradation than CS, exhibiting the following trend: SF-30 > CS > SF-20 > SF-10. For the higher heat treatment temperature (SR, 200 °C), retrogradation consistently exceeded that of CS across all treatment times and progressively increased with longer heat treatment times. After the starch paste cools, the aggregation of amylose molecules tends to promote retrogradation, contributing to the formation of ordered structures [41]. The lower the DHT temperature and the shorter the duration, the weaker the starch retrogradation, resulting in a more stable starch paste system.

### 3.6. Digestive Properties

The digested starch content of CS after DHT is presented in Table 1. As shown in Table 1, the RDS and SDS contents of heat-treated CS showed a significant increase (*p* < 0.05), while the RS content exhibited a significant decrease (*p* < 0.05) compared to CS. The RDS content increased as the heat treatment temperature rose, whereas the RS content decreased. Additionally, the RDS content demonstrated a significant increase (*p* < 0.05) with longer treatment times at constant temperatures. In the lower heat treatment temperature group (SB, 100 °C), the RS content followed the order SB-20 > SB-10 > SB-30, with the highest RS content recorded at 100 °C after 20 min (SB-20). At medium and higher heat treatment temperatures (SF, 160 °C; SR, 200 °C), the RS content decreased progressively with extended heat treatment times, following the trend SF/SR-10 > SF/SR-20 > SF/SR-30. DHT increases the surface area available to digestive enzymes by enlarging holes and fissures on the surface of starch granules, which speeds up the digestion of starch [15]. Furthermore, DHT disrupts the starch molecular structure, weakening the intermolecular bonds in starch chains. This disruption reduces crystalline regions, rendering starch more amenable to enzymatic digestion. Additionally, DHT promotes the gelatinization and regeneration of chestnut starch, making gelatinized starch more accessible to digestive enzymes [42], thereby further increasing the rate of digestion and the RDS content.

The hydrolysis curves of chestnut starch after DHT are shown in Figure 2H. The data indicate that the hydrolysis rate of CS increased in the SB, SF, and SR groups compared to CS, with higher heat treatment temperatures leading to faster digestion rates. This trend aligns with the observed increase in RDS content. The digestion characteristic parameters of chestnut starch after DHT are summarized in Table 1. Notably, the hydrolysis curve fit coefficients (R^2^) for all groups exceeded 0.95, demonstrating a high degree of reliability and strong model fit. After DHT, the percentage of endpoint starch hydrolysis (C∞) increased in all treatment groups, consistent with the observed rise in hydrolysis rates. Additionally, C∞ increased with heat treatment time at medium and higher temperatures (SF-160 °C; SR-200 °C). The temperature and duration of the heat treatment also had an impact on the digestion rate constant (K). Specifically, K increased with heat treatment time at both lower (SB, 100 °C) and higher (SR, 200 °C) temperatures. Within the medium heat treatment temperature group (SF, 160 °C), the order of K was SF-30 > SF-10 > SF-20. The estimated glycemic index (eGI) is a measure of the rate at which starch releases glucose under simulated gastrointestinal conditions. The eGI of starch showed an increase after DHT. Higher heat treatment temperatures led to higher eGI values. Similarly, eGI rose with heat treatment time in both medium (SF) and higher (SR) heat treatment groups, consistent with the trends observed for RDS and K.

### 3.7. Thermal Property

Differential scanning calorimetry (DSC) is a thermal analysis technique commonly employed to investigate starch’s thermal properties. The DSC curves of chestnut starch before and after thermal processing are shown in Figure 2I. As the heat treatment temperature increased, the peak positions of the DSC curves for each group of chestnut starch gradually shifted to the left. Table 2 displays the chestnut starch’s thermal property parameters. After heat treatment, there was a significant (*p* < 0.05) decrease in the onset gelatinization temperature (To), peak gelatinization temperature (Tp), termination gelatinization temperature (Tc), and enthalpy of gelatinization (∆H) in comparison to CS. To, Tp, Tc, and ∆H all showed substantial decreases at the same heat treatment time (*p* < 0.05) as the heat treatment temperature increased, similar findings have been reported for rice starch [42]. A declining trend as the heat treatment duration increased in the SB group at the same heat treatment temperature, but there were no discernible changes in the Tp, Tc, or ∆H. In the SF group, as the heat treatment time increased, Tp and Tc decreased, To and ∆H significantly decreased (*p* < 0.05), and R showed no significant variation. In the SR group, as the heat treatment time increased, there was no significant change in To, while Tp, Tc, ∆H, and R all exhibited decreasing trends. DHT may cause the starch molecular chains to break down and the microcrystalline structure to be disrupted, producing short-chain starch molecules that are easier to dissolve and paste, which lowers the gelatinization temperature [43]. Additionally, DHT can disrupt the intramolecular or intermolecular hydrogen bonds within starch, thereby promoting the swelling of starch granules and leading to a reduction in the gelatinization onset temperature [44]. Research by Lei et al. on starch has shown that the disruption of the ordered structure of starch during the dry heat treatment (DHT) process can also result in a decrease in the gelatinization temperature [45]. Furthermore, the temperature and duration of DHT affect the degree of molecular destruction, which results in different decreases in the enthalpy and gelatinization temperature of chestnut starch molecules.

### 3.8. Scanning Electron Microscopy (SEM) Analysis

**Figure 3:**
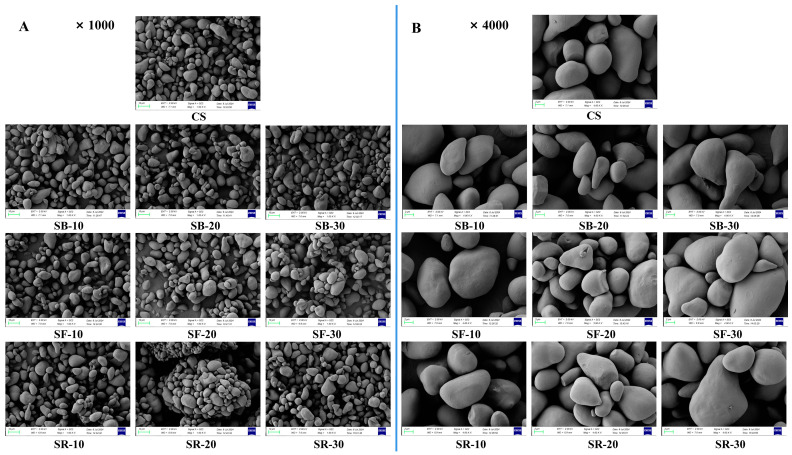
Scanning electron microscope (SEM) images at ×1000 magnification (**A**); SEM images at ×4000 magnification (**B**) of chestnut starch after dry heat treatment. CS, chestnut starch; SB-10/20/30, simulate boiling (100 °C) for 10 min, 20 min, and 30 min; SF-10/20/30, simulate frying (160 °C) for 10 min, 20 min, and 30 min; and SR-10/20/30, simulate roasting (200 °C) for 10 min, 20 min, and 30 min.

Scanning electron microscopy (SEM) was used to analyze the morphology of CS after DHT, as shown in Figure 3A,B at magnifications of 1000× and 4000×, respectively. The CS granules exhibited multiple morphologies, including round, elliptic, triangular, and irregular forms. Their surfaces were initially smooth, lacking observable pores or folds. After DHT, the surfaces of the CS granules in all groups became partially uneven, characterized by slight depressions, folds, small air holes, and cracks. However, DHT did not induce substantial morphological alterations in the starch granules [46]. The gelatinization of the starch granule progressively spread from its surface to its core as the treatment temperature and duration increased. Simultaneously, the surfaces became rough, which might have caused starch granules to aggregate and create bigger structures [15].

### 3.9. Fourier Transform Infrared (FT-IR) Spectra Analysis

**Figure 4:**
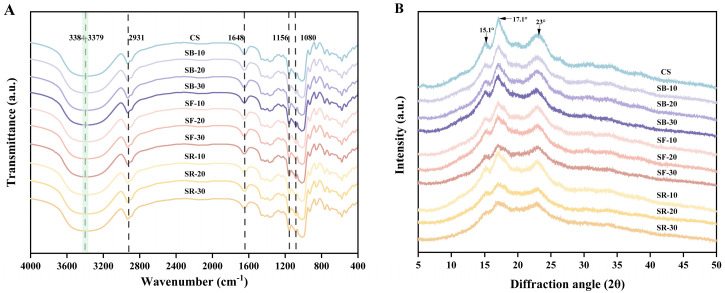
Fourier transform infrared (FT-IR) spectra (**A**); X-ray diffraction (XRD) analysis (**B**) of chestnut starch after dry heat treatment. CS, chestnut starch; SB-10/20/30, simulate boiling (100 °C) for 10 min, 20 min, and 30 min; SF-10/20/30, simulate frying (160 °C) for 10 min, 20 min, and 30 min; and SR-10/20/30, simulate roasting (200 °C) for 10 min, 20 min, and 30 min.

The FT-IR spectra of CS after DHT are depicted in Figure 4A. All samples exhibited similar spectral profiles, indicating that DHT did not disrupt the chemical bonds in starch molecules [11]. The broad band in the range of 3600–3000 cm^−1^ was attributed to the stretching vibrations of hydroxyl groups and hydrogen bonding interactions between starch molecules. Absorption bands in the 3000–2800 cm^−1^ range corresponded to the stretching vibrations of methyl and methylene groups in starch. The absorption bands in the 1200–1000 cm^−1^ region arose from overlapping contributions of ring vibrations, stretching modes of C–OH side groups, and vibrations of C–O–C glycosidic bonds [47]. The absorption bands in the 800–400 cm^−1^ range primarily reflected the skeletal vibrations of glucopyranose rings. The stretching characteristics of single-bond CH2 groups were observed at 2931 cm^−1^, while the absorption band near 1648 cm^−1^ originated from the bending vibration of hydroxyl groups in bound water within starch [48]. The modes at 1156 cm^−1^ and 1080 cm^−1^ were associated with the stretching of C–O single bonds in glucose rings [49]. After DHT, the O–H stretching vibration band in the 3600–3000 cm^−1^ region shifted to higher wavenumbers. This phenomenon may result from the breakage of intermolecular or intramolecular hydrogen bonds in starch due to DHT, which increased the freedom of O–H bonds and elevated their vibrational frequencies, thereby shifting the absorption bands to higher wavenumbers [50]. Additionally, DHT reduced the moisture content in starch, weakening hydrogen bonding interactions between water molecules and starch hydroxyl groups, further contributing to the increased vibrational frequency of O–H bonds. Furthermore, DHT may induce changes in starch crystallinity (supported by the results in Section 3.10) or molecular alignment, altering the vibrational environment of O–H bonds and leading to the observed peak shifts.

### 3.10. Crystal Structure

The XRD pattern of chestnut starch after DHT is shown in Figure 4B. CS exhibits diffraction peaks at 2θ values of 15.1°, 17.1°, and 23°. Table 2 indicates that the starches’ relative crystallinity (RC) decreased significantly (*p* < 0.05) after DHT and exhibited an overall downward trend as the temperature of DHT rose. Among the groups, the RC of the SB group decreased significantly (*p* < 0.05) with extended heat treatment time, while the RC of the SF and SR groups exhibited minimal changes. The reduction in RC suggests increased molecular disorder and the disruption of starch hydrogen bonds [51]. Different thermal processing temperatures and times exert varying effects on starch’s relative crystallinity. The decline in RC may be associated with damage to crystalline regions, partial granule pasting, and double-helix rearrangements during dry heat [16]. Additionally, alterations in the double helix caused by DHT may disrupt the RC or alter the crystalline ordering of starch, contributing to reduced crystallinity [52].

### 3.11. Correlation Analysis

**Figure 5:**
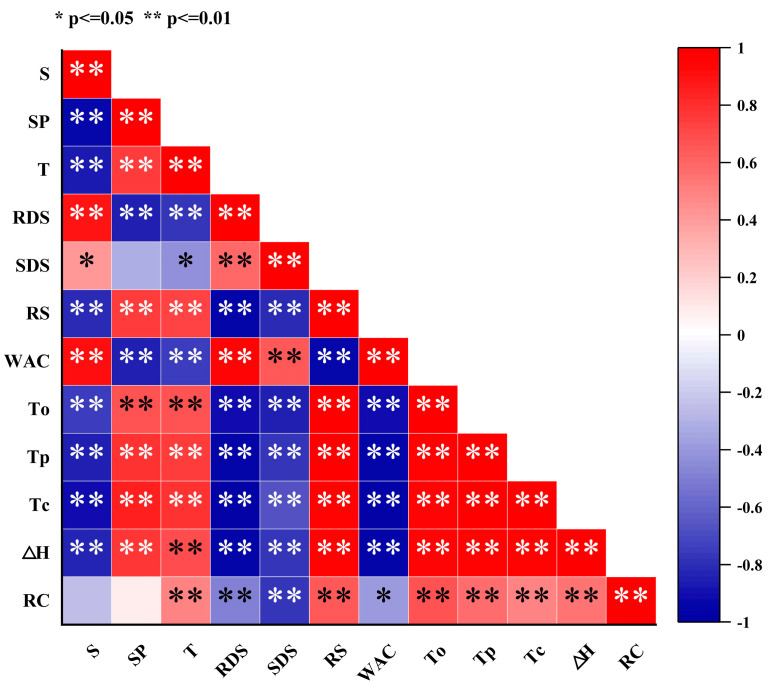
Correlation analysis of the physicochemical and structural properties of chestnut starch after dry heat treatment. S, solubility; SP, swelling power; T, transmittance; RDS, rapidly digestible starch; SDS, slowly digestible starch; RS: resistant starch; WAC, water absorption capacity; To, onset temperature; Tp, peak temperature; Tc, conclusion temperature; ΔH, gelatinization enthalpy; and RC, relative crystallinity.

Heat treatment significantly affects the structure and physicochemical properties of starch, resulting in a positive correlation between solubility and water absorption capacity, while showing a negative correlation with swelling power and transmittance (Figure 5). This phenomenon can be attributed to the partial breakage of hydrogen bonds between starch molecules and the swelling and partial disintegration of starch granules during heat treatment, facilitating the interactions between starch and water molecules and thereby increasing solubility and water absorption capacity. However, the disintegration of starch granules reduces the swelling power of the system, while the weakening of intermolecular interactions leads to decreased transmittance. Heat treatment also significantly influences the pasting and digestibility characteristics of starch, as evidenced by decreases in gelatinization temperature and enthalpy, an increase in RDS content, and a reduction in RS content. This is primarily due to the structural disruption within starch granules, involving the partial breakage of hydrogen bonds between molecular chains and the reduction or rearrangement of crystalline regions, thereby lowering the energy and temperature required for gelatinization. Additionally, heat treatment facilitates starch hydrolysis by increasing enzyme accessibility, promoting the formation of RDS, while RS decreases due to crystalline region damage. This phenomenon explains the negative correlation between gelatinization temperature/enthalpy and RDS content, and the positive correlation with RS content. Heat treatment leads to decreases in the relative crystallinity, gelatinization temperature, and enthalpy of starch, with relative crystallinity positively correlated with gelatinization temperature and enthalpy. This is because heat treatment disrupts the ordered crystalline structure within starch granules, reducing crystalline regions and causing hydrogen bond breakage or rearrangement between molecular chains, thereby decreasing the stability of starch and the energy and temperature required for gelatinization. The reduction in crystallinity indicates a more disordered internal structure of starch granules, which absorb water, swell, and disintegrate more easily during gelatinization, resulting in lower gelatinization enthalpy and temperature.

## 4. Conclusions

This study examines changes in the physicochemical and structural properties of CS under varying DHT temperatures and durations, offering data to support its physical modification. The physicochemical investigation revealed that when temperatures rose, the S and WAC of CS increased, which facilitates rapid dissolution or water absorption in foods. A reduction in the gelatinization temperature after DHT promotes the faster expansion and gelatinization of starch at lower temperatures. Additionally, the RDS content and digestion rate of chestnut starch increase with higher heat treatment temperatures during DHT, enabling rapid energy provision for short-term needs. Structurally, DHT disrupts starch granules, resulting in reduced RC. These structural changes lead to increased solubility, improved digestion rates, and reduced gelatinization temperatures, providing pathways for enhancing starch quality. This study provides data supporting the practical application and modifiability of starch-based foods during processing. However, given the observed increase in resistant starch (RS) and the estimated glycemic index (eGI), incorporating active ingredients such as flavonoids in future studies could enhance this functional property, making it more suitable for consumption by special populations.

## Figures and Tables

**Table 1:** Parameters of the digestive properties of chestnut starch after DHT.

Processing Method	RDS (%)	SDS (%)	RS (%)	C∞ (%)	K (min^−1^)	R^2^	AUC	HI	eGI
CS	18.52 ± 0.38 ^j^	24.46 ± 0.04 ^g^	57.02 ± 0.35 ^a^	45.13	0.0213	0.9872	3458	31.50	57.00
SB-10	21.49 ± 0.34 ^i^	35.02 ± 0.23 ^d^	43.49 ± 0.53 ^c^	60.94	0.0176	0.9813	4263	38.83	61.03
SB-20	22.97 ± 0.37 ^h^	30.95 ± 0.15 ^f^	46.08 ± 0.40 ^b^	55.68	0.0208	0.9803	4221	38.45	60.82
SB-30	24.57 ± 0.16 ^g^	32.07 ± 0.32 ^e^	43.35 ± 0.46 ^c^	56.88	0.0222	0.9751	4444	40.48	61.93
SF-10	27.91 ± 0.05 ^f^	41.06 ± 0.19 ^c^	31.03 ± 0.23 ^d^	71.75	0.0219	0.9942	5575	50.78	67.59
SF-20	30.65 ± 0.67 ^e^	43.67 ± 1.81 ^b^	25.68 ± 2.35 ^e^	77.96	0.0208	0.9849	5916	53.88	69.29
SF-30	32.71 ± 0.92 ^d^	46.64 ± 0.16 ^a^	20.65 ± 1.07 ^f^	80.52	0.0232	0.9893	6405	58.34	71.74
SR-10	51.70 ± 0.53 ^b^	40.17 ± 0.28 ^c^	8.13 ± 0.33 ^g^	88.38	0.0356	0.9797	8156	74.29	80.50
SR-20	50.54 ± 0.39 ^c^	42.68 ± 0.21 ^b^	6.78 ± 0.54 ^gh^	90.60	0.0359	0.9850	8381	76.34	81.62
SR-30	53.35 ± 0.39 ^a^	40.24 ± 0.20 ^c^	6.40 ± 0.51 ^h^	93.74	0.0396	0.9975	8900	81.07	84.22

Values are presented as means ± SD; the different letters after the values are significantly different (*p* < 0.05). CS, chestnut starch; SB-10/20/30, simulate boiling (100 °C) for 10 min, 20 min, and 30 min; SF-10/20/30, simulate frying (160 °C) for 10 min, 20 min, and 30 min; SR-10/20/30, simulate roasting (200 °C) for 10 min, 20 min, and 30 min; RDS, rapidly digestible starch; SDS, slowly digestible starch; RS, resistant starch; C∞, endpoint starch hydrolysis; K, digestion rate constant; R^2^; hydrolysis curve fit coefficients; AUC, area under the curve; HI, hydrolysis index; and eGI, estimated glycemic index.

**Table 2:** Thermal properties and RC parameters of chestnut starch after DHT.

Processing Method	To (°C)	Tp (°C)	Tc (°C)	∆H (J/g)	R (°C)	RC (%)
CS	59.61 ± 0.11 ^a^	65.37 ± 0.09 ^a^	71.15 ± 0.15 ^a^	11.51 ± 0.32 ^a^	11.54 ± 0.15 ^de^	26.02 ± 0.09 ^a^
SB-10	58.33 ± 0.05 ^b^	64.38 ± 0.07 ^b^	70.35 ± 0.11 ^b^	10.68 ± 0.26 ^b^	12.02 ± 0.15 ^cd^	19.56 ± 0.07 ^b^
SB-20	58.01 ± 0.21 ^bc^	64.30 ± 0.61 ^b^	70.36 ± 0.23 ^b^	10.85 ± 0.21 ^b^	12.35 ± 0.18 ^bc^	18.07 ± 0.08 ^c^
SB-30	57.71 ± 0.20 ^d^	64.07 ± 0.04 ^b^	70.32 ± 0.25 ^b^	10.93 ± 0.23 ^b^	12.61 ± 0.08 ^b^	16.38 ± 0.04 ^e^
SF-10	54.97 ± 0.15 ^e^	61.57 ± 0.16 ^c^	68.17 ± 0.24 ^c^	9.14 ± 0.16 ^d^	13.19 ± 0.11 ^a^	17.02 ± 0.13 ^d^
SF-20	54.33 ± 0.20 ^f^	60.80 ± 0.68 ^d^	68.01 ± 0.58 ^c^	9.61 ± 0.18 ^c^	13.68 ± 0.53 ^a^	16.34 ± 0.14 ^e^
SF-30	53.76 ± 0.57 ^g^	60.24 ± 0.48 ^d^	66.96 ± 0.52 ^d^	8.42 ± 0.42 ^e^	13.20 ± 0.56 ^a^	16.16 ± 0.43 ^e^
SR-10	52.44 ± 0.27 ^h^	58.27 ± 0.43 ^e^	64.45 ± 0.27 ^e^	7.36 ± 0.10 ^f^	12.01 ± 0.21 ^cd^	16.97 ± 0.23 ^d^
SR-20	52.45 ± 0.09 ^h^	57.66 ± 0.24 ^ef^	63.60 ± 0.34 ^f^	7.33 ± 0.30 ^f^	11.15 ± 0.27 ^e^	16.94 ± 0.08 ^d^
SR-30	52.46 ± 0.20 ^h^	57.14 ± 0.17 ^f^	62.75 ± 0.30 ^g^	7.23 ± 0.13 ^f^	10.29 ± 0.20 ^f^	16.86 ± 0.03 ^d^

Values are presented as means ± SD; the different letters after the values are significantly different (*p* < 0.05). CS, chestnut starch; SB-10/20/30, simulate boiling (100 °C) for 10 min, 20 min, and 30 min; SF-10/20/30, simulate frying (160 °C) for 10 min, 20 min, and 30 min; SR-10/20/30, simulate roasting (200 °C) for 10 min, 20 min, and 30 min; To, onset temperature; Tp, peak temperature; Tc, conclusion temperature; ΔH, gelatinization enthalpy; R, gelatinization temperature range; and RC, relative crystallinity.

## Data Availability

The data will be made available upon request.
